# Prevalence of excessive screen time and its association with developmental delay in children aged <5 years: A population-based cross-sectional study in India

**DOI:** 10.1371/journal.pone.0254102

**Published:** 2021-07-06

**Authors:** Samya Varadarajan, Akila Govindarajan Venguidesvarane, Karthik Narayanan Ramaswamy, Muthukumar Rajamohan, Murugesan Krupa, Sathiasekaran Bernard Winfred Christadoss

**Affiliations:** 1 Department of Community Medicine, Sri Ramachandra Institute of Higher Education and Research, SRIHER, Chennai, India; 2 Department of Pediatric Critical Care, Rainbow Children’s Hospital, Chennai, India; 3 Department of Speech, Language and Hearing, Sri Ramachandra Institute of Higher Education and Research, SRIHER, Chennai, India; ESIC Medical College & PGIMSR, INDIA

## Abstract

The global growth of electronic media usage among children has caused concerns regarding screen time (ST) impact on child development. No previous population-based studies have evaluated ST and child development in India. This study aimed to determine the burden of ST, associated sociodemographic factors, and its impact on domains of child development. A population-based cross-sectional study was conducted in the field practice area of rural and urban health centers in Tamil Nadu, India. A total of 718 children (396 rural and 322 urban) were selected, using a cluster random sampling method. ST estimates were obtained from parents/guardian after a 7-day observation period. The Communication DEALL Developmental Checklist was used to assess child development. The mean ST was 2.39 hours/day (95% confidence interval [CI]: 2.23–2.54), and the prevalence of excessive ST was 73% (95% CI: 69.2–76.8). Excessive ST was significantly associated with the mothers’ ST, screen usage at bedtime, birth order (in children < 2 years), and attending school (in children ≥ 2 years). Increased ST was significantly associated with developmental delay, in particular, in the domains of language acquisition and communication. In children aged ≥ 2 years, a delay in ≥ 3 domains was associated with ST (adjusted odds ratio [AOR] = 17.75, 95% CI: 5.04–62.49, p < 0.001), as was language delay (AOR = 52.92, 95% CI: 12.33–227.21, p < 0.001). In children aged < 2 years, a delay in ≥ 2 domains was associated with ST (AOR = 16.79, 95% CI: 2.26–124.4, p < 0.001), as was language delay (AOR = 20.93, 95% CI: 2.68–163.32, p < 0.01). A very high prevalence of excessive ST was identified, with a significant association with developmental delay in children. There is an urgent need to include education on ST limits at the primary healthcare level.

## Introduction

Electronic media usage has seen explosive growth globally. Children today are exposed to screen-based entertainment and information, which has become an integral part of their lives. In the United States, children between the ages of 0–8 years spend more than 2 hours per day on screens [1]; in western India, the mean screen time (ST) is 2.7 hours among children aged 2–6 years [2].

Due to the malleability of children’s social and intellectual development in the early years, it is critical to evaluate the impact of ST during this period [3]. Excessive ST has been associated with language and motor skill developmental delay, [3,4] in addition to its psychosocial impact [5]. According to the American Association of Pediatrics guidelines, children aged <2 years should not have any ST, and those aged 2–5 years should be limited to 1 hour of high-quality education programs per day, monitored by their caregivers [6]. The Canadian Pediatric Society issued similar guidelines in 2017 [7], as did the World Health Organization (WHO) in 2019, proposing ST limits in children aged < 5 years [8]. However, these guidelines have been criticized as not being evidence-based [9], and the findings on ST impact have been inconsistent in systematic reviews.

While some evidence on the impact of ST on development is available from high-income Western countries, that from low-and middle-income countries is lacking [10]. The assessment of ST in the existing studies is mostly based on caregivers’ recollection, which is subject to bias. In addition, most research on ST is based on data from hospital, clinic/well-child immunization and pre-school records, without any population-based studies assessing child development at home. The coronavirus disease 2019 pandemic has led to global school closures and a shift to online education. Studies are required for evidence-based guidelines, aimed at establishing healthy ST limits for children [11].

This study aimed to determine the prevalence of excessive ST, associated sociodemographic factors, and its effects on various domains of child development among the population in India.

## Materials and methods

### Study design and population

A population-based cross-sectional study was conducted in the field practice and demonstration areas of rural and urban health and training centers of the Medical College and Research Institute in South India. Based on a previous study [2], a minimum sample size of 360 was required for a mean ST of 2.7 ± 1.7 hours, and a relative precision of 6.5. The design effect of 2 was considered, and the final sample size was set at 720. The sampling frame of the Rural Health and Training Center consists of 1218 children aged < 5 years, distributed in 9 panchayats (divisions). In comparison, the Urban Health and Training Center area has 1356 children in 19 divisions. A cumulative list of the population aged < 5 years was created and the villages of the panchayats were identified as clusters in rural areas, as well as the sub-divisional regions in urban areas, with a probability proportional to size. From each cluster, 30 children were selected. Children aged < 6 months, parents who refused consent, children with known intellectual disabilities, congenital anomalies, and genetic conditions such as trisomy 21 were excluded. In households with multiple children fitting the criteria, only one child was randomly included in this study.

### Study variables and measurements

After obtaining written informed consent from the child’s mother/guardian data on background demographic characteristics, living conditions, socioeconomic status [12], and parents’ education were obtained.

#### Screen time

ST was estimated by requesting the mother/primary caregiver to observe the child over a period of 7 days and record their ST in a diary, which was later collected through re-visitation. Health inspectors and public health nurses were instructed to follow up with the parents/caregivers over the telephone to ensure that ST was documented daily. For children attending playschool/balwadi/daycare, we sent a letter to the teachers through the parents requesting them to document the children’s ST. The mean amount of time spent on screens (in hours) during the 7-day observation period was considered as the ST of the child. Details of the type of screen used, age at first exposure, and weekday/weekend frequency of screen use were also collected. Excessive ST for children aged between 6 months to 2 years was defined as any ST per day; for those aged 2–5 years, it was defined as >1 hour of ST per day, as per the 2019 WHO guidelines [9].

#### Developmental assessment

Child development was assessed through the Communication DEALL Developmental Checklist [13,14], which is a validated tool for child assessment in India. This tool covers eight domains including gross motor, fine motor, receptive language, expressive language, activities of daily living, cognitive skills, and social and emotional development. Each domain includes 36 items, which makes a total of 288 items. Children aged 0–72 months were categorized into a developmental hierarchy at 6-month intervals, thereby creating 12 groups. Each group contained three skills on the checklist and each skill was scored on a 5-point scale.

0 Not acquired
1 Acquired but lost
2 Acquired but inconsistently present
3 Acquired and consistently present, but only in specific situations
4 Acquired and consistently present in all situations

A child was classified as having a developmental delay in a domain if two or more skills were not acquired in the child’s developmental hierarchy. Developmental delay was categorized as delay in any one domain, in two or more, or three or more domains. A separate analysis was conducted for receptive or expressive language delay, language or social interaction delay, and communication skills delay.

### Data collection and quality control measures

Data were collected from January to May 2019. A team of two members, a speech-language pathologist, and the principal or co-investigator visited each house. Since ST details were not collected on the same day, the speech-language pathologist was blinded to the ST estimates of the child.

### Ethical approval

Institutional Ethics Committee approval was obtained before the commencement of this study. The Institutional Ethics Committee of Sri Ramachandra Institute of Higher Education and Research, Chennai, India, approved all experimental protocols (Ref No: IEC-NI/18/SEP/66/53). This study was conducted in accordance with the relevant guidelines and regulations from the ethics committee and the Declaration of Helsinki. Children who were identified as having significant developmental delay were referred for further evaluation and management.

### Consent to participate

Because this study included minors, written informed consent was obtained from the parents/guardians. Informed consent was translated into the local language and validated by language experts.

### Statistical analysis

Statistical Package for the Social Sciences (SPSS), version 16 (IBM Corporation, Somers, NY, USA), was used for data entry and analysis. The background variables were expressed as frequencies and percentages. The prevalence of excessive ST and 95% confidence intervals (CIs) were calculated. Factors associated with excessive ST were examined, using the chi-square test; the corresponding odds ratios (ORs) and 95% CIs were calculated. Statistical significance was set at a two-sided p-value of <0.05. A logistic regression model using the enter method was created for children in two groups (6–23 months and 24–60 months) with ST as an independent variable and development in each domain as a dependent variable; adjusted odds ratios (AORs) were calculated to account for the impact of probable confounders.

## Results and discussion

A total of 718 children were included in this study, including 396 and 322 children from urban and rural areas, respectively. Approximately half of the children were male (49.7%), and the mean age was 34.7 ± 15.8 months. Most children (61.3%) were a part of a nuclear family. The sociodemographic characteristics of the study population are shown in Table 1.

**Table 1:** Sociodemographic characteristics and screen time details of study participants.

S No	Background variables	N	%
1	**Sex**		
	Males	358	49.7
	Females	360	50.3
2	**Age**		
	<18 months	118	16.4
	18–23 months	77	10.7
	24 months and older	523	72.8
3	**Type of Family**		
	Nuclear	440	61.3
	Extended nuclear	228	31.8
	Joint	46	6.4
	Others	4	0.6
4	**Modified Prasad’s Socio-economic status** [12]		
	Class 1	131	18.2
	Class 2	228	31.8
	Class 3	231	32.2
	Class 4	123	17.1
	Class 5	5	0.7
5	**Mothers education**		
	Postgraduate	76	10.6
	Graduate/diploma	190	26.5
	Higher Secondary school	136	18.9
	High School	204	28.4
	Middle School	88	12.3
	Primary School	16	2.2
	Illiterate	8	1.1
6	**Mothers occupation**		
	Homemaker	609	84.8
	Employed	108	15.2
7	**Mother’s/caregiver’s ST**		
	<2 hours	282	39.3
	>2 hours	436	60.7
8	**Whether the child going to playschool/balwadi/daycare (N = 523)**		
	Yes	333	63.8
	No	190	36.2
9	**Age at first exposure to screen**		
	Not exposed	23	3.2
	<6 months	61	8.5
	7–12 months	435	60.8
	13–18 months	42	5.9
	19–23 months	99	13.8
	More than 24 months	56	7.8
10	**Screen time on weekends**		
	Increases	173	24.2
	Decreases	19	2.7
	Is the same as on weekdays	523	72.8
11.	**A circumstance when the screen is given to the child**		
	While feeding/eating	529	74.4
	Parents are at household chores	412	57.9
	As a reward	108	15.2
	To calm the child	280	39.3
	Distract the child	205	28.8
	At outdoor (restaurants, functions, etc)	40	5.6
	Traveling by bus, car, train, etc	34	4.8
	During illness	23	3.2
	At bedtime	222	31.2

The mean overall ST was 2.39 hours per day (95% CI: 2.23–2.54). The children were mostly exposed to smartphones and televisions (Table 2).

**Table 2:** Details of screen time.

Screen type	6–23 months		24–60 months		Total	
	Mean (95% CI) (hours)	SD (hours)	Mean (95% CI) (hours)	SD (hours)	Mean (95% CI) (hours)	SD (hours)
**Smart phone**	0.74 (0.61–0.87)	0.96	1.35 (1.23–1.47)	1.45	1.2 (1.1–1.3)	1.37
**TV**	0.72 (0.58–0.86)	1	1.59 (1.47–1.71)	1.43	1.36 (1.26–1.46)	1.39
**Total ST**	1.26 (1.04–1.48)	1.55	2.8 (2.6–2.9)	2.23	2.39 (2.23–2.54)	2.18

Most children (72.8%) had similar ST on weekdays and weekends; a total of 24.2% and 2.7% of the children had increased and decreased exposure during the weekends, respectively. The screen was accessible in the bedroom in 21.6% of cases. Only 4.9% of parents (n = 35) had implemented ST rules for their children. Among them, 5.9% did not allow any ST, 76.5% and 17.6% allowed for 1 and 2 hours of ST per day, respectively. Regarding the implementation of these rules, 34.3% of parents declared they were always able to implement them, 45.7% implemented them sometimes, and 20.0% could not implement them at all.

The rates of excessive ST among children aged <2 years and those aged ≥ 2 years were 73.3% (95% CI: 67.1–79.5) and 73.0% (95% CI: 69.2–76.8), respectively. Excessive ST was not associated with residence area (rural/urban), socioeconomic status, family type, or education level of the mother or caregiver. However, a statistically significant association was found between excessive ST and children attending balwadi for children aged >2 years (p < 0.05), as well as the birth order of children aged < 2 years (p < 0.05). Excessive ST was significantly associated with screen use at bedtime and the ST of the mothers among children of all age groups (Table 3).

**Table 3:** Factors associated with screen time.

		Children aged 24–60 months					Children aged 6–23 months				
S no	Background variable	Excess ST N (%)	Normal ST N (%)	OR	95% CI	p	Excess ST N (%)	Nil ST N (%)	OR	95% CI	p
1	**Place of residence**										
	Rural	158(69)	71 (31)	0.69	0.47–1.02	0.66	28 (30.1)	65 (69.9)	0.83	0.45–1.51	0.53
	Urban	224 (76.2)	70 (23.8)				35 (34.3)	67 (65.7)			
2	**Gender**										
	Male	194 (74.6)	66 (25.4)	1.17	0.8–1.73	0.42	30 (30.6)	68 (69.4)	0.86	0.47–1.56	0.611
	Female	188 (71.5)	75 (23.8)				33 (34)	64 (66)			
3	**Birth order**										
	1	222 (72.5)	84 (27.5)	0.94	0.64–1.3	0.764	43 (38.7)	68 (61.3)	2.02	1.08–3.8	0.029
	2 or more	160 (73.7)	57 (26.3)				20 (23.8)	64 (76.2)			
4	**Going to balwadi/playschool**										
	Yes	256 (76.9)	77 (23.1)	1.69	1.1–2.5	0.009					
	No	126 (66.3)	64 (33.7)								
5	**Type of family**										
	Nuclear	245 (73.1)	90 (26.9)	1.01	0.68–1.52	0.94	36 (34.3)	69 (65.7)	1.22	0.67–2.23	0.523
	Extended/joint	137 (72.9)	51 (27.1)				27 (30)	63 (70)			
6	**Modified BG prasad’s SES**										
	Class 1	69 (69.7)	30 (30.3)			0.49	13 (40.6)	19 (59.4)			0.55
	Class 2	127 (76)	40 (24)				21 (34.4)	40 (65.6)			
	Class 3	120 (74.5)	41 (25.5)				21 (30)	49 (70)			
	Class 4 &5	66 (68.8)	30 (31.2)				8 (25)	24 (75)			
7	**Mother’s education**										
	Middle school and above	365 (72.6)	138 (27.4)	0.47	0.14–1.62	0.21	63 (33)	128 (67)	0.67	0.61–0.74	0.163
	Primary or below	17 (85)	3 (15)				0 (0)	4 (100)			
8	**Mothers screen time**										
	>2 hours	273 (83)	56 (170)	2.45	1.83–3.28	0.000	74 (69.2)	33 (30.8)	1.62	1.14–2.31	0.006
	<2 hours	113 (58.2)	81 (41.8)				44 (50)	44 (50)			
8	**Screens used at bedtime**										
	Yes	150 (96.2)	6 (3.8)	14.5	6.3–33.8	0.000	1 (25)	3 (75)	0.69	0.07–6.80	0.044
	No	232 (63.2)	135 (36.8)	5			62 (32.5)	129 (67.5)			

### Child development

Among the children aged < 2 years, 1.5% (n = 3) had a delay in gross motor development, 2.1% (n = 4) in fine motor development, 3.6% (n = 7) in activities of daily living, 11.8% (n = 23) in expressive language, 6.2% (n = 12) in receptive language, 4.6% (n = 9) in social interaction, and 3.6% (n = 7) in emotional development. Among children aged ≥ 2 years, the corresponding rates were 1.5% (n = 8), 3.6% (n = 19), 3.4% (n = 18), 24.5% (n = 128), 14.1% (n = 74), 8.6% (n = 45), and 2.9% (n = 15), respectively. In addition, 7.1% (n = 37) of children aged >2 years had cognitive delay.

Bivariate analysis of child development in any domain and background variables showed that excessive ST was significantly associated with developmental delay (Table 4). This analysis was performed separately for two age groups, as the definition of excessive ST differed between them. A logistic regression model using the enter method was performed, and the ORs were adjusted for the following confounders: residence location, sex, birth order, family type, socioeconomic status, and mother’s education. The AORs were 7.64 (95% CI: 1.67–34.85) and 19.28 (95% CI: 6.65–55.59) for children aged < 2 years and for those aged ≥ 2 years, respectively. Excessive ST was the only factor associated with developmental delay.

**Table 4:** Developmental delay domains and their associated factors.

		Children aged 24–60 months					Children aged 6–23 months				
S no	Background variable	Delay 1 or more domains N	Normal development N	OR	95% CI	p	Delay 1 or more domains N	Normal development N	OR	95% CI	p
1	**Place of residence**										
	Rural	65	164	0.88	0.61–1.29	0.52	16	77	0.97	0.46–2.03	0.935
	Urban	91	203				18	84			
2	**Gender**										
	Male	83	177	1.22	0.84–1.78	0.29	76	22	0.48	0.23–1.05	0.06
	Female	73	190				85	12			
3	**Birth order**										
	2 or more	140	227	5.22	3.58–7.62	<0.0001	10	74	0.49	0.22–1.09	0.08
	1	79	77				24	87			
4	**Going to balwadi/playschool**										
	Yes	93	240	0.78	0.53–1.15	0.21					
	No	63	127								
5	**Type of family**										
	Nuclear	103	135	1.13	0.76–1.15	0.54	14	91	0.53	0.25–1.14	0.11
	Extended/joint	53	232				20	70			
6	**Modified BG Prasad’s SES**										
	Class 1	34	65			0.748	4	28			0.714
	Class 2	48	119				13	48			
	Class 3	47	114				11	59			
	Class 4 &5	27	69				6	26			
7	**Mother’s education**										
	Middle school and above	150	353	0.99	0.37–2.62	0.98	34	157	1.97	0.1–37.48	0.65
	Primary or below	6	14				0	4			
8	**Screen time excess**										
	Yes	151	231	17.78	7.11–44.23	<0.0001	32	111	7.21	1.66–21.25	0.008
	No	5	136				2	50			

### ST and child development

Tables 5 and 6 present the association between ST and child development domains. Increasing ST was associated with increased odds of development delay, in particular, in the language and communication domains. For children aged < 2 years, an ST usage was significantly associated with any one domain of developmental delay (Table 4). When ST was more than 1 hour, there was significant delay in language domains. Similar results were observed in children aged ≥ 2 years when ST was more than 1 hour. In binary logistic regression analysis, the OR was adjusted for probable confounders such as socioeconomic status, mother’s education level and occupation, sex of the child, and place of residence. AORs were higher for both age groups. The wide CI was likely due to the small number of children with ST of < 1 hour in the ≥ 2-year age group and no ST in the < 2 years group with developmental delay.

**Table 5:** Association between screen time in hours (STh) and child development among children aged 24–60 months.

S NO	DOMAIN	STh	DELAY N	NORMAL N	OR	95% CI	AOR	95% CI
1	Any one domain	>2	123	104	32.43*	12.8–82.18	37.61*	14.48–97.69
		1–2	28	127	5.99*	2.25–16.01	6.6*	2.44–17.86
		<1	5	136				
2	2 or more domains	>2	82	104	35.74*	10.98–116.33	39.97*	12.05–132
		1–2	18	127	6.43*	1.85–22.34	6.63*	1.89–23.27
		<1	3	136				
3	3 or more domains	>2	30	104	13.08*	3.88–44.03	17.75*	5.04–62.497
		1–2	9	127	3.21	0.85–12.13	3.49	0.91–13.296
		<1	3	136				
4	Receptive or expressive language delay	>2	72	104	47.08*	11.29–196.33	52.92*	12.33–227.21
		1–2	14	127	27.5*	1.67–33.64	8.22*	1.798–37.598
		<1	2	136				
5	Language or social interaction delay	>2	82	104	53.62*	12.89–223.09	61.69*	14.38–264.61
		1–2	14	127	7.5*	1.67–33.64	8.33*	1.82–38.12
		<1	2	136				
6	Any communication skill delay	>2	101	104	33.02*	11.77–92.61	38.02*	13.22–109.37
		1–2	19	127	5.09*	1.69–15.36	5.62*	1.83–17.22
			4	136				

*p<0.05.

**Table 6:** Association between screen time in hours (STh) and child development among children aged 6–23 months.

S NO	DOMAIN	STh	DELAY N	NORMAL N	OR	95% CI	AOR	95% CI
1	Any one domain	>1	25	43	14.53*	3.25–64.94	35.74*	5.74–222.67
		0–1	7	68	2.57	0.51–12.92	3.93	0.614–251.17
		0	2	50				
2	2 or more domains	>1	11	43	6.395*	1.34–30.46	16.79*	2.26–124.44
		0–1	4	68	1.47	0.26–8.34	2.79	0.36–21.89
		0	2	50				
3	3 or more domains	>1	5	43	5.81	0.65–51.71	9.61	0.85–108.58
		0–1	3	68	2.21	0.22–21.84	4.04	0.32–50.798
		0	1	50				
4	Any communication skill delay	>1	20	43	23.26*	2.99–180.53	135.58*	6.26–2935.47
		0–1	5	68	3.68	0.42–32.46	8.64	0.45–167.52
		0	1	50				
5	Language or social interaction delay	>1	18	43	20.93*	2.68–163.32		
		0–1	2	68	1.47	0.13–16.67		
		0	1	50				

*p<0.05.

This study, conducted in a rural and urban field practice area at a medical college, aimed to identify the impact of ST on child development. The mean ST in children under 5 years of age was 2.39 ± 2.18 hours per day (95% CI: 2.23–2.54). Excessive ST was significantly associated with attending school (in children aged ≥ 2 years), birth order (in children aged < 2 years), screen availability at bedtime, and ST of the mothers. Increased ST was significantly associated with developmental delay, in particular, in the language and communication domains.

In our study, the narrow CI of the mean ST indicates good internal validity and sufficient sample size. The mean ST for children aged < 2years and those aged 2–5 years was 1.26 hours and 2.8 hours, respectively. Similar findings were observed in a study conducted in Western India, which reported the mean ST of 2.7 hours among children aged 2–5 years [2]; in addition, Ruangdaraganon et al. reported 1.21 hours for 1-year-olds and 1.69 hours for 2-year-olds [15]. However, studies conducted in Melbourne [16,17], Europe [18], and Korea [3] had lower mean ST estimates (1.8 hours, 1.32 hours, 1.21 hours, respectively). These differences could be attributed to the different study locations as well as better parental control. An extensive literature review revealed a lack of studies on ST per device, although ST estimates for mobile phone [19–21], television, and computer usage have been reported [17,22–26]. Our study has documented the separate mean usage of television and smartphones, which were 1.36 hours and 1.2 hours, respectively.

Our results revealed that 89% of the children were exposed to at least one type of screen before the recommended age of 24 months, whereas a study in Korea by Chang et al. reported that only 65% and 31.3% of children were exposed to television and smartphones, respectively [27]. This evidence shows that children in India are exposed to screens early, which requires further observation. Most caregivers (72.8%) reported no difference in ST duration between weekdays and weekends. This finding is in contrast to those in studies conducted by Chang [27], Jago [20], and Georgia et al. [18], which showed increased ST on weekends. These differences may be associated with study settings, as well as sociocultural differences.

Excessive ST in children was not correlated with socioeconomic status, place of residence, or the mother’s education level in our study. In comparison, Cheng [28] and Fulton et al. [25] found an inverse association between ST and socioeconomic status, and Cheng [28] and Emond et al. [24] found an association between excessive ST and parent/caregiver educational levels. Mothers’ ST was found to be associated with high ST in children in our study. Primary healthcare providers should inform parents/caregivers about the impact of their ST on that of the children in their care.

Moreover, an increase in ST was significantly associated with a delay in the development of language, communication, and social interaction among children; these findings are consistent with those of a study conducted by Madigan et al. [26], where excessive ST was associated with child developmental delay. Lin [3] and Wu et al. [19] also found positive associations between ST and delays in cognitive, functional, language, and motor development; meanwhile, van den Heuvel et al. [29] showed a significant association between cell phone use and expressive speech delay. The first 5 years are critical to a child’s development, and a healthy environment is crucial to their upbringing. In this study, screens were mostly introduced to children during meals or when the caregiver was doing chores, suggesting that the purpose of ST is not always educational, and that parental monitoring may be ineffective. Such use of devices leads to reduced social interaction with caregivers [30–32] and developmental delay in terms of communication skills.

### Limitations

The causal relationship between ST and developmental delay could not be established due to the study’s cross-sectional nature. Since the ST details were collected prospectively, the parents/caregivers could have modified the children’s screen exposure, reducing the mean ST. This study was conducted in a small part of India, within rural and urban areas in Tamil Nadu. Therefore, the mean values of ST may not be representative of those observed elsewhere in the state or country and cannot be generalized due to differences in ethnicity, culture, and language (mother tongue). The tool used for child development assessment has only been validated as a screening method in the Indian setting and may not be used for diagnosis. However, since there is a significant association between ST and developmental delay, it is important to limit children’s ST. This study only evaluated the negative effects of ST on child development. Although high-quality educational videos may benefit fine motor skill [33], and language [34] and vocabulary acquisition [35], in particular, when combined with parental interaction, the overall evidence suggests that the damage caused by screens outweighs the benefits [36].

### Strengths

This is the first population-based study in India to assess the association between ST and the development of children aged <5 years. The assessment was performed by a speech-language pathologist in the comfortable environment of the child’s home, which ensured better cooperation and assessment. All domains of child development were examined to ensure a better understanding of the association between ST and each domain. Recall bias was reduced as the data regarding ST were collected prospectively after observing the child for a week.

## Conclusions

This study found a significant association between ST and child development. Since the prevalence of excessive ST is extremely high, limiting ST in children is essential to ensure healthy development. Even though high-quality videos may support skill acquisition, it is difficult to ensure supervised ST; parents should be advised to prevent leaving their children unsupervised with screens. The WHO ST limits should be considered when implementing reproductive and child health programs as an essential part of childcare services. This aim can be achieved by involving primary care physicians and paramedical workers in rural and urban primary healthcare centers to educate parents and caregivers on limiting ST. Future studies should focus on effective methods to reduce and maintain low ST in children.

## Supporting information

S1 File Screen time data file. (XLSX)
